# Vascular and Cardiovascular Outcomes of Smoking Cessation and Switching to Electronic Cigarette Use: Protocol for a Systematic Review of Prospective Studies

**DOI:** 10.2196/74949

**Published:** 2025-09-29

**Authors:** Giusy Rita Maria La Rosa, Jacqueline Yu, Giulio Geraci, Davide Capodanno, Takao Ohki, Jacob George, Riccardo Polosa

**Affiliations:** 1 Department of Clinical and Experimental Medicine University of Catania Catania Italy; 2 University of Dundee Medical School Dundee United Kingdom; 3 Faculty of Medicine and Surgery Kore University of Enna Enna Italy; 4 Azienda Ospedaliero-Universitaria Policlinico "Gaspare Rodolico-San Marco" University of Catania Catania Italy; 5 Department of Surgery Jikei University Hospital Tokyo Japan; 6 Center of Excellence for the Acceleration of Harm Reduction University of Catania Catania Italy

**Keywords:** cardiovascular disease, e-cigarettes, smoking cessation, stroke, vascular function

## Abstract

**Background:**

Despite the well-documented adverse health effects of tobacco smoking, it remains prevalent worldwide. Chronic tobacco smoking is associated with significant cardiovascular effects, including an increased risk of cardiovascular disease and vascular dysfunction. In recent years, the use of electronic cigarettes (ECs) has increased, with many former tobacco cigarette smokers switching to ECs to aid in smoking cessation. To date, no systematic review has synthesized prospective evidence on key vascular and cardiovascular outcomes among adult smokers who quit or switch to ECs, with the specific aim of identifying early and clinically meaningful indicators of vascular recovery.

**Objective:**

This systematic review aims to summarize the current prospective studies on the vascular effects of switching to exclusive EC use and the overall effects of smoking cessation on regular tobacco cigarette smokers. The review will focus, in particular, on early indicators of vascular improvement, such as measures of vascular function, including arterial stiffness and endothelial function, as well as on heart rate, blood pressure, nonfatal myocardial infarction, nonfatal stroke, sudden cardiovascular death, and acute heart failure.

**Methods:**

The review will be reported following the PRISMA (Preferred Reporting Items for Systematic Reviews and Meta-Analyses) guidelines. A comprehensive search was performed in PubMed and Scopus on January 12, 2025, without restrictions on the publication date. Studies will be included if they focus on cardiovascular outcomes, with study designs, including clinical trials (randomized controlled trials, observational studies), prospective cohort studies, and case-control studies. Studies involving regular smokers, aged 18 years and older, and those reporting on smoking cessation or switching to ECs will be considered. Data will be extracted using a standardized form and synthesized qualitatively. Risk of bias will be assessed using Joanna Briggs Institute’s critical appraisal tools, with results informing sensitivity analyses and interpretation.

**Results:**

As of January 2025, the preliminary search retrieved 1479 records across PubMed and Scopus. At the time of this protocol’s publication, the first manuscript on vascular function outcomes was completed in July 2025 and is currently under peer review. Each subsequent review will include an updated search before completion.

**Conclusions:**

This review will provide a comprehensive landscape of current literature on the long-term vascular effects of smoking cessation and switching to exclusive EC use. The findings will help identify early indicators of vascular damage reversal, gaps in the current evidence base, and inform future research directions. By disseminating the results through peer-reviewed journals, conferences, and digital platforms, the review aims to enhance the understanding of smoking cessation’s impact on vascular health and support public health efforts in reducing tobacco-related harm.

**Trial Registration:**

PROSPERO CRD420251016878; https://www.crd.york.ac.uk/PROSPERO/view/CRD420251016878

**International Registered Report Identifier (IRRID):**

PRR1-10.2196/74949

## Introduction

### Background

Tobacco smoking prevalence remains high worldwide and causes over 7 million deaths annually from smoking-related diseases [1,2]. Chronic tobacco smoking has significant cardiovascular effects impacting vascular function, leading to increased risk of cardiovascular disease and sudden cardiac death [3-5]. Conversely, stopping smoking has been shown to significantly reduce the risks of cardiovascular events [6,7], and guidelines strongly recommend smoking cessation for primary and secondary prevention of cardiovascular disease [8-10]. Efforts to promote smoking cessation have intensified, with research showing that success rates can double or triple when counseling is combined with pharmacotherapy such as varenicline [11], cytisine [12], bupropion [13], and nicotine replacement therapy [14]. Additionally, Cochrane systematic reviews, meta-analyses, and umbrella reviews suggest that electronic cigarettes (ECs) may also be effective for smoking cessation [15,16]. The US Food and Drug Administration and UK’s National Institute for Health and Care Excellence (NICE) recognize ECs as less harmful than conventional smoking and a useful adjunct for smoking cessation [17,18].

### Gaps in the Existing Literature

Most evidence linking smoking cessation to improved cardiovascular outcomes and vascular function comes from cross-sectional studies, which cannot establish causality. In addition, prior reviews often aggregate studies with highly heterogeneous designs, populations, and outcome measures, and many focus only on short-term or acute effects following limited EC or nicotine exposure. These limitations hinder our understanding of the long-term vascular consequences of smoking cessation or switching to exclusive EC use. Furthermore, a key limitation in evaluating smoking cessation and harm reduction strategies is the lack of early, sensitive, and clinically meaningful end points. This gap has slowed progress in understanding the full health impact of cessation therapies and reduced-risk nicotine or tobacco products. Advancing regulatory science and clinical research in this area requires identifying early indicators of biological and physiological recovery. Yet, this search remains challenging. Our understanding of early smoking–induced pathophysiological changes is incomplete, and sensitive, clinically relevant end points for early health impact assessment are still poorly defined. Although functional vascular end points are widely used, they often lack the sensitivity to capture subtle, early-stage changes in recent quitters. Specifically, no systematic review to date has synthesized prospective evidence focused on key vascular and cardiovascular end points and incident cardiovascular events among adult smokers who either quit or switch to ECs in order to detect early and clinically significant end points of vascular damage reversal.

### Study Objectives

To address these limitations, this systematic review aims to synthesize prospective evidence on the vascular and cardiovascular outcomes associated with smoking cessation and switching to exclusive EC use in order to identify early and clinically relevant indicators of vascular recovery. The review will examine changes in vascular function—including pulse wave velocity (PWV), intima-media thickness (IMT), and flow-mediated dilatation (FMD)—as well as cardiovascular indicators such as blood pressure and heart rate, and the incidence of major events, including nonfatal myocardial infarction, nonfatal stroke, sudden cardiovascular death, and acute heart failure. In addition, it will explore methodological patterns, assess the quality of available studies, and highlight areas for future research.

## Methods

The systematic review will be reported following the principles outlined in PRISMA (Preferred Reporting Items for Systematic Reviews and Meta-Analyses) [19] and has been registered in PROSPERO (registration CRD420251016878).

### Search Strategy

A thorough literature review was performed in January 12, 2025, by using PubMed and Scopus databases. Search queries have been designed to identify relevant studies in each database. Details of the search strings for each database are provided in Table 1. To identify additional literature, 2 reviewers will examine the reference lists of the included studies. Medical experts will also be consulted to ensure that no recent relevant studies have been overlooked. The search will be conducted without any restrictions on publication date. Only studies published in English will be considered.

**Table 1:** Search strategy for each database.

Database	Search query	Date
PubMed	(((“smoking”[MeSH Terms] OR “smoking”[Title/Abstract] OR “smok*”[Title/Abstract] OR “cigarette”[Title/Abstract]) AND (“cessation”[Title/Abstract] OR “quit*”[Title/Abstract] OR “abstinence”[Title/Abstract] OR “stop*”[Title/Abstract])) OR (“ex-smokers”[Title/Abstract] OR “former smokers”[Title/Abstract])) AND (“myocardial infarction”[MeSH Terms] OR “myocardial infarction”[Title/Abstract] OR “ischemic stroke”[MeSH Terms] OR “ischemic stroke”[Title/Abstract] OR “stroke”[MeSH Terms] OR “stroke”[Title/Abstract] OR hemorrhagic stroke[MeSH Terms] OR hemorrhagic stroke[Title/Abstract] OR “Major Adverse Cardiac Events (MACE)”[Title/Abstract] OR “Blood Pressure”[MeSH Terms] OR “Blood Pressure”[Title/Abstract] OR “Arterial Pressure” [MeSH Terms] OR “Arterial Pressure”[Title/Abstract] OR “Heart Rate” [MeSH Terms] OR “Heart Rate”[Title/Abstract] OR “Vascular Stiffness” [MeSH Terms] OR “Vascular Stiffness”[Title/Abstract] OR “Pulse Wave Velocity”[Title/Abstract] OR “Pulse Wave Analysis”[MeSH Terms] OR “Pulse Wave Analysis”[Title/Abstract] OR “Augmentation index”[Title/Abstract] OR AI@75[Title/Abstract] OR AI75[Title/Abstract] OR “Endothelial function”[Title/Abstract] OR “flow mediated dilatation”[Title/Abstract] OR “forearm venous occlusion”[Title/Abstract] OR “plethysmography”[MeSH Terms] OR plethysmography[Title/Abstract] OR “EndoPAT”[Title/Abstract] OR “peripheral arterial tonometry”[Title/Abstract] OR “microscopic angioscopy”[MeSH Terms] OR “microscopic angioscopy” [Title/Abstract] OR capillaroscopy[Title/Abstract]) AND (“follow-up studies”[MeSH Terms] OR follow up studies[Text Word] OR “Cohort Studies”[MeSH Terms] OR “Cohort Studies”[Text Word] OR “prospective studies”[MeSH Terms] OR “prospective studies”[Text Word])	January 12, 2025
Scopus	(“smoking” OR “smok*” OR “cigarette”) AND (“cessation” OR “quit*” OR “abstinence” OR “stop*” OR “ex-smokers” OR “former smokers”) AND (“myocardial infarction” OR “ischemic stroke” OR “stroke” OR hemorrhagic AND stroke OR “Major Adverse Cardiac Events (MACE)” OR “Blood Pressure” OR “Arterial Pressure” OR “Heart Rate” OR “Vascular Stiffness” OR “Pulse Wave Velocity” OR “Pulse Wave Analysis” OR “Augmentation index” OR “flow mediated dilatation” OR “plethysmography” OR “peripheral arterial tonometry” OR “microscopic angioscopy” OR capillaroscopy) AND (“follow-up studies” OR “Cohort Studies” OR “prospective studies” OR “longitudinal studies” OR “longitudinal”)	January 12, 2025

### Study Selection

The studies will be selected based on the following inclusion and exclusion criteria.

#### Inclusion Criteria

The following studies are included.

1. Studies with the following end points: vascular function (eg, PWV, augmentation index, cardio-ankle vascular index, IMT, photoplethysmography, stiffness index, FMD), nonfatal myocardial infarction, nonfatal stroke, cardiovascular death, acute heart failure, heart rate/heart rate variability, blood pressure.
2. Study design: clinical trials (randomized controlled trials), parallel observational or interventional, prospective cohort studies, and case-control studies.
3. Participants: patients aged 18 years and older who are classified as regular smokers according to each study’s own definition. We chose not to apply a strict quantitative threshold at the inclusion stage, as many primary studies do not provide standardized or detailed metrics of smoking exposure (eg, cigarettes per day, duration, pack-years). Instead, we will extract and tabulate all available quantitative indicators of smoking behavior and describe the variability across studies. Where feasible, subgroup or sensitivity analyses will be conducted based on different levels or definitions of exposure to assess their impact on the overall findings.
4. Study populations with conditions defined as risk factors for cardiovascular disease will be included: rheumatoid arthritis, diabetes mellitus, dyslipidemia, and hypertension.
5. Studies on smoking cessation or switching studies (they may include dual EC and tobacco cigarette users).
6. Data (quantitative or narrative) on the effect of smoking cessation on the outcome.

#### Exclusion Criteria

The following studies are excluded.

1. Preclinical studies, retrospective cohort, cross-sectional, case series/reports.
2. Review studies, conference papers/studies not peer-reviewed.
3. Studies with fewer than 30 participants and without a sample size calculation or justification.
4. Length of study less than 1 week.
5. Switching studies using other smokeless tobacco aside from ECs (ie, snus/shisha).

To ensure alignment with the review’s objective, we will exclude studies where persistent dual use (ie, simultaneous EC and tobacco cigarette use) is not clearly separated from exclusive EC use. If a study includes participants who transitioned from dual use to exclusive EC use, data will be included only if the outcomes are reported after the point of exclusive use and the time frame is explicitly defined. Additionally, when outcomes are stratified by biochemical verification (eg, CO levels), we will consider those subgroups separately. This approach is intended to minimize the risk of confounding and improve interpretability of cardiovascular effects attributed to exclusive EC use.

Two independent reviewers will screen each title and abstract to identify studies for full-text review. Studies that appear suitable for inclusion or cannot be clearly excluded based on the title and abstract will be carried forward for further assessment. The records and duplicates will be managed using EndNote (version 21; Thomson Reuters). Each remaining full-text study will then be independently evaluated by the 2 reviewers to determine its eligibility. Any disagreements will be resolved through discussion and consensus. Studies meeting the predefined inclusion criteria will be selected for the qualitative/quantitative synthesis.

### Data Extraction and Synthesis of Findings

Data extraction will be performed using a standardized form specifically designed for this purpose. The relevant data, including the lead author's name (surname, first name), publication year, country, study design, mean age of the cohort(s), percentage of male and female participants, sample size, study population, comorbidities (if applicable), prior smoking history (minimum cigarettes per day), prior smoking history (pack years), whether dual use of e-cigarettes and smoking is allowed (yes/no), whether other concomitant nicotine products are allowed (yes/no), follow-up duration (in weeks), vascular and cardiovascular end point, and main findings (focused solely on the effects of abstinence from cigarette smoking on vascular outcomes, with quantitative data provided where available or summarized narratively), will be systematically recorded and organized in a table. Potential limitations and conclusions of the studies will also be noted. In the qualitative synthesis, emphasis will be placed on the clinical impact of smoking abstinence on vascular outcomes.

If sufficient and comparable data are available, a meta-analysis will be performed. The primary measures of effect will be the odds ratio with a corresponding 95% CI for dichotomous outcomes and the mean difference with a 95% CI for continuous outcomes. Due to potential variations in study populations, interventions, and outcome definitions across trials, a random-effects model will be employed to account for potential heterogeneity among studies. The meta-analysis will be conducted using the *STATA/BE* (version 17; StataCorp LT) statistical package. Heterogeneity among the included studies will be assessed using the *I*² statistic, with an *I*² value >50% considered indicative of substantial heterogeneity. Potential publication bias will be assessed visually using funnel plots. If a meta-analysis is not feasible due to substantial heterogeneity among studies, a narrative synthesis will be conducted.

### Risk of Bias Assessment

Two authors will independently assess the methodological quality of the included studies, using the Joanna Briggs Institute’s critical appraisal tools tailored to each specific study design [20]. Each checklist includes a set of items covering core domains such as participant selection, exposure and outcome measurement, control of confounding, and the appropriateness of statistical analysis. Any disagreements will be resolved through discussion to reach a consensus or by consulting a third author if necessary. Risk of bias assessments will be presented in a tabular or graphical format, with responses recorded for each item per study. A narrative synthesis will accompany the tables, summarizing methodological quality across studies grouped by study design. The risk of bias assessments will inform the synthesis and interpretation of results. In particular, sensitivity analyses will be conducted—quantitative or narrative—by excluding studies presenting multiple methodological limitations as identified through the Joanna Briggs Institute’s checklists. Additionally, we will transparently report how the quality of the included studies influences the level of confidence in the findings, especially in the narrative synthesis. This approach will help highlight areas of greater methodological robustness versus vulnerability.

### Findings Reporting

To ensure a focused and comprehensive analysis, each major end point category (ie, vascular function, heart rate/blood pressure, and major cardiovascular outcomes) will be addressed in separate publications. These domains are methodologically and clinically distinct, often involving different measures, statistical models, and implications for harm reduction. Rather than merging all the findings into a single, overly complex manuscript, this approach is intended to enhance clarity, support in-depth interpretation, and serve both clinical and research audiences more effectively. To prevent fragmentation, each paper will include a concise overview of the full systematic review protocol and clearly explain the rationale behind the separate reporting. Publications will be cross-referenced to allow readers to easily access related findings. A final synthesis manuscript will integrate all the results and discuss their broader clinical and public health implications.

## Results

As of January 12, 2025, the preliminary literature search has been completed for the database searches, with the following results: PubMed (n=1126), Scopus (n=696), duplicates (n=343), and a total of 1479 records. At the time of publication of this protocol, the first manuscript, focusing on vascular function outcomes, was completed in July 2025 and is currently under peer review. The full series of reviews, covering all outcome domains, is scheduled for completion by December 2026. To ensure the inclusion of the most up-to-date evidence, the search strategy will be rerun and updated prior to the finalization of each individual review.

## Discussion

This protocol outlines the methodology for a systematic review aimed at synthesizing prospective evidence on the vascular and cardiovascular outcomes associated with smoking cessation and switching to exclusive EC use. By focusing on early indicators of vascular recovery, this review aims to support progress in both regulatory science and clinical research, enabling a more accurate and timely assessment of the long-term cardiovascular effects of smoking cessation and switching to alternative nicotine products.

A clearer understanding of the direct effects of stopping smoking on vascular health and cardiovascular risk factors in people who smoke has been sought in a number of reviews [21,22]. However, their conclusions have been limited by the heterogeneity of the included studies, which predominantly consist of cross-sectional designs. Cross-sectional studies provide only a snapshot of cardiovascular health at a single time point and are inherently limited in their ability to establish causality. Given the dynamic nature of vascular changes following smoking cessation, longitudinal studies are essential for capturing sustained improvements in endothelial function, arterial stiffness, and overall cardiovascular health.

Additionally, the studies included in these review studies often vary in sample characteristics, smoking history, follow-up duration, and outcome measures, contributing to inconsistent findings. Furthermore, a major limitation of these reviews is their reliance on studies that primarily assess the vascular effects of smoking cessation and switching to ECs in hyperacute settings, rather than focusing on long-term outcomes. Acute studies, which typically measure vascular parameters within hours or days following single-dose or limited-dose exposure, provide valuable insights into immediate physiological responses to nicotine and other aerosol components. However, these short-term changes do not necessarily reflect long-term cardiovascular risks or benefits [23]. Moreover, acute studies may either overestimate or underestimate the actual impact of EC use and smoking cessation, as they fail to account for adaptation mechanisms, compensatory physiological responses, or cumulative exposure effects over time. A more comprehensive understanding requires longitudinal studies that evaluate sustained vascular changes and their clinical relevance over extended periods. Switching studies, which examine the cardiovascular effects of transitioning from combustible cigarettes to ECs, are particularly susceptible to these biases [24-30]. Many of these studies are limited by small sample sizes, short follow-up periods, or lack of adequate control groups [24-27]. We designed this systematic review with these limitations in mind, focusing exclusively on prospective studies with adequate follow-up periods to obtain more realistic and reliable data on the cardiovascular effects of smoking cessation and switching to ECs.

Vascular end points such as PWV, IMT, and FMD were selected due to their well-established roles as surrogate markers of cardiovascular risk. More specifically, PWV reflects arterial stiffness and is strongly associated with future cardiovascular events, including myocardial infarction, stroke, and cardiovascular mortality [31,32]. IMT is a structural indicator of early atherosclerosis and correlates with coronary and cerebrovascular outcomes, particularly in individuals younger than 65 years [33]. FMD assesses endothelial function and has demonstrated predictive value for cardiovascular events; a meta-analysis showed that each 1% improvement in FMD is associated with a 13% reduction in major adverse cardiac events [34]. These markers capture distinct yet complementary aspects of vascular health—mechanical (PWV), structural (IMT), and functional (FMD)—and are sensitive to early changes that may occur following smoking cessation or switching to ECs. Including these end points enhances the capacity of this review to detect early vascular improvements and evaluate their clinical relevance, particularly in the short-to-medium term, where traditional hard end points may not yet be observable.

A key strength of this protocol is the rigorous focus on prospective study designs, which enhances the potential to draw temporally relevant and clinically meaningful inferences. The review’s scope is broad enough to capture diverse vascular end points, yet specific in its inclusion criteria to minimize heterogeneity. A potential limitation is the exclusion of retrospective and cross-sectional studies, which may contain useful but less robust observational data. However, this decision was made to prioritize internal validity and reduce the risk of bias.

Upon completion, the findings from this systematic review will be submitted for publication in peer-reviewed journals, presented at international conferences, and disseminated through academic networks focused on tobacco harm reduction and cardiovascular prevention. The results may help inform clinical guidelines, policy discussions, and the design of future longitudinal studies evaluating the health impact of alternative nicotine products.

In conclusion, this systematic review protocol outlines a structured and rigorous approach to evaluating the prospective evidence on the vascular and cardiovascular effects of smoking cessation and switching to exclusive EC use. By focusing on high-quality study designs and clinically relevant early end points, the review aims to produce a meaningful synthesis of the available literature, identify gaps for future research, and contribute to the ongoing discussion around harm reduction and cardiovascular risk mitigation in smokers. The findings will support researchers, clinicians, and policymakers in understanding the long-term impact of these behavioral transitions on vascular health.

## Abbreviations

EC: electronic cigarette
FMD: flow-mediated dilatation
IMT: intima-media thickness
NICE: National Institute for Health and Care Excellence
PRISMA: Preferred Reporting Items for Systematic Reviews and Meta-Analyses
PWV: pulse wave velocity

## Data Availability

Data sharing is not applicable to this study, as no data sets were generated or analyzed during this study.
